# ANCA-associated vasculitis overlaps with systemic sclerosis: a case report and literature review

**DOI:** 10.1186/s40001-021-00500-2

**Published:** 2021-03-31

**Authors:** Rui Wu, Jiang Su, Yu-rong Zou, Jing Zhu

**Affiliations:** 1 Department of Rheumatology and Immunology, Sichuan Provincial People’s Hospital, University of Electronic Science and Technology of China, No.32 The First Ring Road West 2, Chengdu, 610072 China; 2 Department of Rheumatology and Immunology, Chinese Academy of Sciences Sichuan Translational Medicine Research Hospital, Chengdu, 610072 China; 3 Department of Nephrology, Sichuan Provincial People’s Hospital, University of Electronic Science and Technology of China, Chengdu, 610072 China

**Keywords:** Systemic sclerosis, Vasculitis, ANCA, ANCA-associated vasculitis

## Abstract

**Background:**

Systemic sclerosis (SSc) and anti-neutrophil cytoplasmic antibody (ANCA)-associated vasculitis (AAV) both affect the kidney and may cause renal failure. Treatment of AAV is dramatically different from that of SSc renal crisis (SRC). Kidney biopsy is not recommended for diagnosing SRC, but it is the only reliable diagnostic method for AAV.

**Case presentation:**

Here, a 49-year-old male patient with diffuse SSc presented with acute renal insufficiency and detectable ANCA with myeloperoxidase-specific antibodies. A renal biopsy revealed necrotizing glomerulonephritis and was consistent with AAV. This finding confirms the existence of AAV and SSc overlap syndrome. The patient was treated with intravenous methylprednisolone, intravenous cyclophosphamide, tandem membrane plasma exchange, and hemodialysis. After treatment, his clinical symptoms remained stable, and his creatinine and C-reactive protein (CRP) levels have remained normalized as of his most recent follow-up after hospital discharge.

**Conclusions:**

AAV can overlap with SSc; although this condition is rare, it is associated with considerable morbidity and mortality. Therefore, it is critical to recognize AAV in the setting of worsening renal function due to SSs and provide appropriate treatment. Several clinical features are suggestive of AAV rather than SRC, but renal biopsy is required for accurate diagnosis.

## Background

Systemic sclerosis (SSc) is a chronic autoimmune disease characterized by progressive thickening of the skin and fibrosing of multiple organs, including the heart, lungs, kidneys, and gastrointestinal tract. The prevalence of SSc ranges from 7 to 489 cases per million population, and its incidence ranges from 0.6 to 122 cases per million population annually [1]. SSc cases are categorized as either limited cutaneous SSc (lcSSc) or diffuse cutaneous SSc (dcSSc) based on the extent of skin involvement [1, 2]. Although the pathogenesis of SSc is not completely understood, it is believed that deregulated production of autoantibodies and cytokines leads to vasculopathy, elevated collagen synthesis, and progressive vascular fibrosis. In assessing SSc renal disease, the most important renal complication is SSc renal crisis (SRC), which is characterized by malignant hypertension, acute renal failure, and microangiopathy [3].

Anti-neutrophil cytoplasmic antibody (ANCA)-associated vasculitis (AAV) refers to a group of autoimmune diseases characterized by necrotizing small vessel vasculitis, comprising granulomatosis with polyangiitis, microscopic polyangiitis, and eosinophilic granuloma with polyangiitis [4]. ANCA-associated glomerulonephritis (AGN), accounting for 64–87.1% of AAV, is typified by kidney involvement, and a kidney biopsy is the current gold standard for confirming its diagnosis [5, 6]. Involvement of the vasculature in ANCA-positive vasculitis following SSc is rare [7]. Acute renal failure occurs in both SRC and AGN, especially in patients with an acute blood pressure increase. Unfortunately, accurately differentiating between SRC and AGN is difficult, making it challenging for clinicians to correctly diagnose patients with these symptoms, and because the treatments for these conditions differ greatly from each other, appropriate diagnosis is critical for achieving a good prognosis [8].

Here, we report the case of a 49-year-old male patient with SSc who developed biopsy-confirmed AAV and presented with acute renal insufficiency, and we review the literature on overlap between SSc and AAV.

## Case presentation

**Fig. 1:**
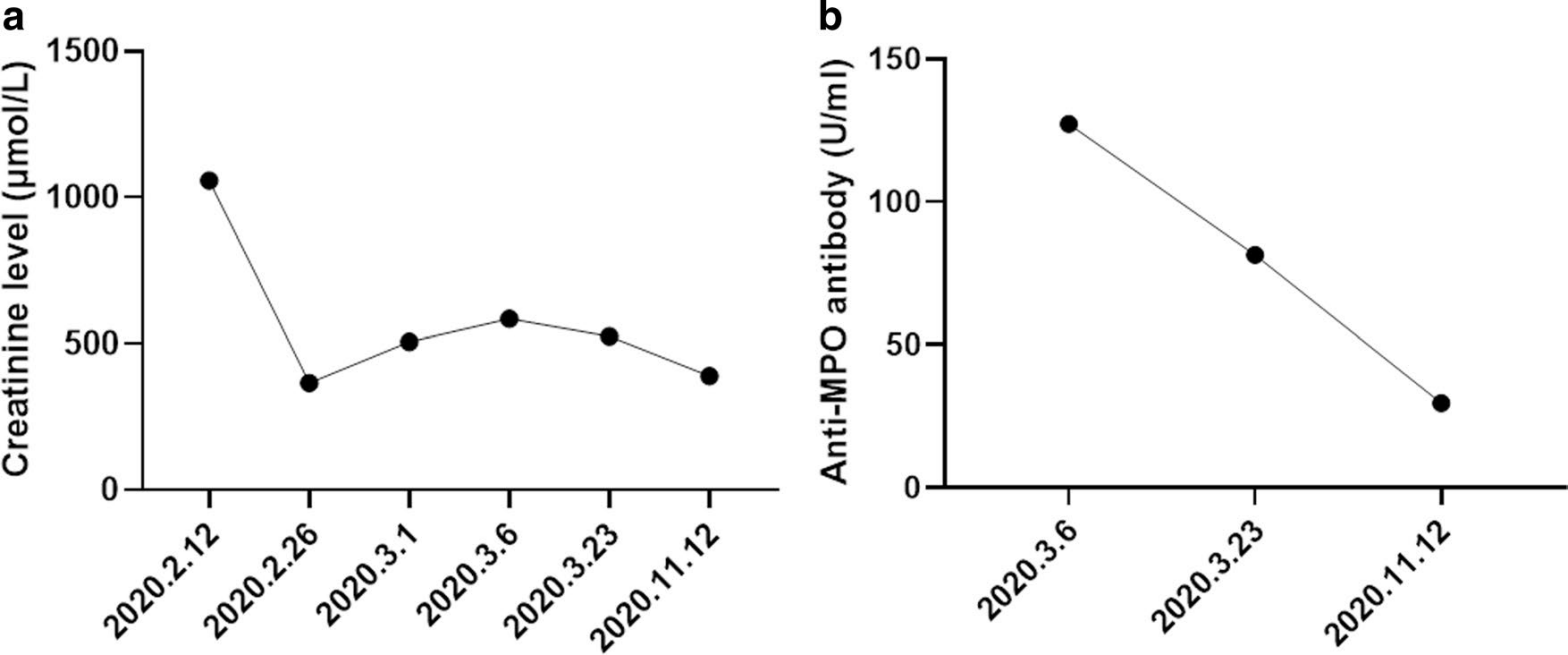
Dynamic changes in the patient’s creatinine and anti-MPO antibody levels. **a**, **b** The patient’s levels of creatinine (**a**) and anti-MPO antibody (**b**) were determined during treatment and follow-up

The patient, a 49-year-old man, was admitted to our hospital for further evaluation of newly diagnosed acute renal insufficiency and highly elevated C-reactive protein (CRP) levels. He had a medical history of Raynaud’s phenomenon and was diagnosed with SSc 4 years previously. At that time, he reported no abnormality of renal function and urinary protein but was not followed up. He did not take any glucocorticoids or penicillamine during this time. In February 2020, 1 month before admission, he visited a doctor in another hospital where he presented with generalized weakness, decreased appetite, oliguria, and bilateral lower extremity swelling. On physical examination, he was found to have elevated blood pressure (176/102 mmHg), elevated levels of blood urea nitrogen (23.17 mmol/L) and creatinine (1057 μmol/L), proteinuria (+++), occult blood (+++), and a 24-h urine protein of 4.65 g. His CRP level was 112 mg/L, erythrocyte sedimentation rate (ESR) was 56 mm/h, and brain natriuretic peptide (BNP) level was 15,452 pg/mL. A kidney ultrasound showed no obvious abnormalities. A chest CT revealed bilateral pleural effusions and patchy shadows in both lung fields. The patient was treated with methylprednisolone (60 mg, daily), antibiotics, an antihypertensive drug, a diuretic treatment, and hemodialysis (three times per week), after which his creatinine level dropped to 366 μmol/L, and his symptoms improved (Fig. 1a). Eight days after these improvements, the methylprednisolone was discontinued following a gradual dose reduction, but the patient’s creatinine level then increased to 506 μmol/L and his CRP level increased to 87 mg/L. At this time (March 6, 2020), the patient was transferred to our hospital. Up until transfer, he was still being treated with hemodialysis.

**Fig. 2:**
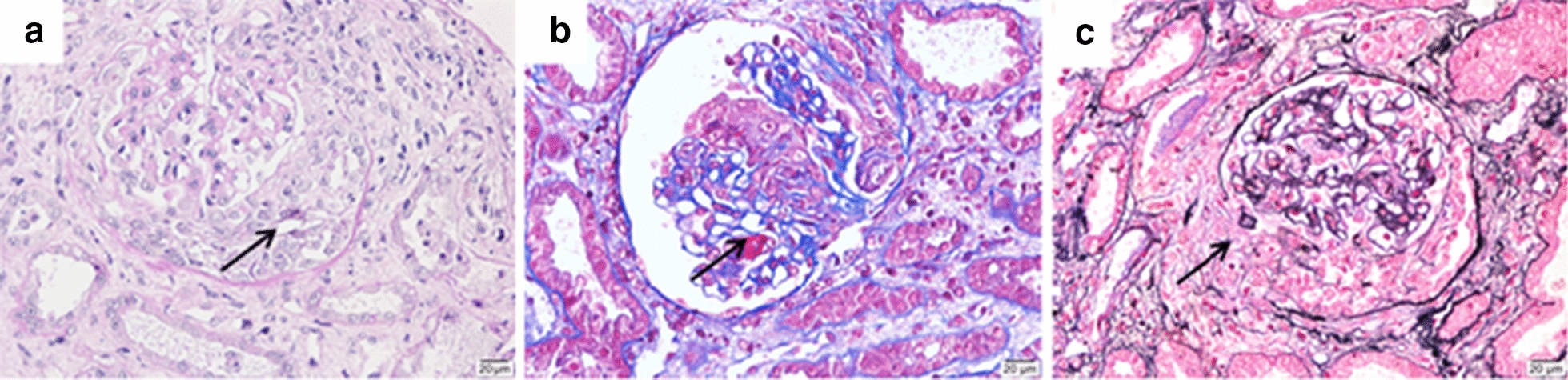
A kidney biopsy revealed cellular crescents in the glomerulus. **a** Cellular crescents (HE, magnification: 400×); **b** fibrinoid necrosis (MASSON, magnification: 400×); **c** cellular crescents and ruptured wall of Bowman’s capsule (PASM, magnification: 400×)

On admission, the patient’s blood pressure was 114/72 mmHg. There were signs of sclerotic skin on his arms that extended to the bilateral elbow joints and on his face, along with evident erythema scattered around his eyelids and cheeks. No obvious edema in the lower extremities was observed, and his liver function was normal. His white blood cell (WBC) count was 10.3 × 10^9^ cells/L, and his hemoglobin (HB) level was 65 g/L, platelet (PLT) count was 147 × 10^9^ cells/L, blood urea nitrogen (BUN) level was 25.62 mmol/L, creatinine level was 584 µmol/L, albumin (ALB) level was 26.8 g/L, and BNP was 132 pg/mL. A urine analysis revealed proteinuria (++), occult blood (+++), and a 24-h urine protein level of 2.42 g. His CRP level was 101 mg/L, ESR was 59 mm/h, serum iron level was 3.6 µmol/L, serum ferritin level was 1520.5 ng/mL, and he had a normal reticulocyte absolute count. We detected high levels of anti-nuclear antibodies (ANA), specifically anti-SSA (+++), anti-SSB (+++), anti-Scl-70 (+++), anti-MPO (127.23 U/mL) (Fig. 1b), and p-ANCA. Cardiac ultrasonography revealed a widened pulmonary artery, with a pulmonary artery systolic pressure of approximately 47 mmHg. Ultrasonography revealed 11.5 × 5.0 cm of his right kidney and 10.4 × 5.2 cm of his left kidney, with a clear boundary between the medulla and cortex. A kidney biopsy revealed 15 glomeruli with formed crescents, of which two were sclerosed, along with six cellular crescents (Fig. 2a, b), three cellular crescents with fibrin deposits, two fibrous crescents, and a ruptured wall of Bowman’s capsule (Fig. 2c); it also showed one segmental fibrinoid necrosis of a capillary loop, a thickened arteriole wall, diffuse interstitial edema, and multiple foci of lymphatic, mononuclear cell, and plasma cell infiltration (about 15%). All these pathological changes resembled the features of crescentic glomerulonephritis and consistent with AAV.

On the basis of the patient’s clinical manifestations, laboratory tests, and kidney biopsy findings, a diagnosis of SSc overlapping AAV was made. The crescentic glomerulonephritis, rather than SRC, was considered to be the likely cause of renal injury. Accordingly, the patient was treated with intravenous methylprednisolone (80 mg, twice daily), intravenous cyclophosphamide (0.2 g, every other day for a total dose of 1.0 g), tandem membrane plasma exchange (3 times), and hemodialysis. During hospitalization, his highest blood pressure was 170/105 mmHg, and anti-hypotensive therapy with nifedipine was initiated. After these therapeutic measures, his creatinine level stabilized around 387–524 μmol/L, 24-h urinary protein decreased to 1.638 g/L, HB increased to 77 g/L, anti-MPO antibody dropped to 81.27 U/mL, and his ESR and CRP level normalized. The patient was discharged on March 27, 2020, with a permanent jugular catheter, which was implanted for regular dialysis. After discharge, he was given oral prednisone (60 mg, qd) plus oral cyclophosphamide (50 mg, qod), and the prednisone was gradually tapered to the dose of 10 mg (qd). The patient has remained under follow-up through the time of writing. During his most recent follow-up in November 2020, he was taking oral prednisone (5 mg, qd) plus cyclophosphamide (50 mg, qod) with a cumulative dose of 6.25 g. The frequency of hemodialysis had been decreased to twice weekly, and the antihypertensive drug had been discontinued. The general condition of the patient had improved; he had a good appetite and no fever, rash, edema, or cough. He had a blood pressure of under 106–128/69–82 mmHg, stable creatinine levels of approximately 389 µmol/L, an anti-MPO antibody concentration of 29.6 U/mL (Fig. 1b), and a normal ESR and CRP level.

The patient has provided informed consent for the publication of this case.

## Discussion and conclusions

Here, we report the case of a patient who had been diagnosed with dcSSc 4 years previously and had chronic kidney insufficiency that progressed with an increase in creatinine levels associated with hypertension and cardiac insufficiency, suggesting the possibility of SRC. However, other clinical features of this patient, such as highly elevated CRP levels and ESR, are not consistent with SRC. The detection of autoimmune antibodies (MPO ANCA) suggested an overlap between SSc and AAV symptoms. A renal biopsy, the gold standard for AGN diagnosis, is mandatory for the diagnosis of SSc overlapping AAV [3]; our findings here confirmed that the crescentic glomerulonephritis rather than the SRC had caused acute renal injury in this case.

Overlap symptoms involving AAV often occur in systemic lupus erythematosus (SLE), rheumatoid arthritis (RA), polymyositis, and SSc. In a retrospective, single-centered study with 247 AAV patients conducted by Martin and his colleagues [9], 28 cases (11.3%) were reported to have a concomitant diagnosis of other autoimmune diseases; the predominant AAV type was renal-limited vasculitis (39%), followed by granulomatosis with polyangiitis (29%), microscopic polyangiitis (25%), and eosinophilic granulomatosis with polyangiitis (7%). Twenty-four cases were positive for ANCA, with the main manifestations of renal (79%), otorhinolaryngologic (43%), and pulmonary and peripheral neuropathy (32%) [9]. The most frequent overlapping connective tissue disease (CTD) was RA (39%), followed by SS and SSc (14%), mixed connective tissue disease (MCTD) (11%), SLE and JIA (7%), and AS and IgG4-RD (4%). Nine patients (32%) were simultaneously diagnosed with AAV and other CTD, but the median interval between both diagnoses in the remainder of cases was 173 months (22–408 months). Sixteen patients (57%) experienced partial or total remission at a median follow-up of 34 months, and four patients (14%) died [9]. In another study conducted by Guibert and his colleagues [10], the frequency and outcome of 106 patients with AAV overlapping with another CTD were analyzed: 15.1% patients had concomitant AAV and another CTD, with RA, SS, and SSc being the most frequent. Compared with the AAV alone group, there were more women, with a higher frequency of venous thrombotic events, and a higher rate of non-renal relapse in the AAV overlapping CTD group [10]. However, no difference between groups was observed for the kidney biopsy and major outcomes [10]. It was suggested that AAV overlapping another CTD is not a rare condition, with RA being the most common overlapping CTD.

The presence of ANCA in SSc is relatively uncommon, with an estimated prevalence of 0–12% [7, 11]. However, although ANCA have been detected in up to 12% of SSc cases, only rare cases present clinical manifestations of AAV. Understanding the clinical significance of ANCA positivity in SSc is of great significance. Different studies have reported conflicting conclusions regarding the main type of ANCA (anti-MPO or anti-PR3) [7, 11]. To improve the diagnostic accuracy, indirect immunofluorescence (IIF) and ELISA should both be used to detect ANCA. Importantly, the clinical manifestations related to vasculitis may be dependent on ANCA titers. Caramaschi et al. found that a patient with a high titer of anti-PR3 antibody had rapidly progressing skin and lung lesions, whereas another patient with a weakly positive detection of anti-MPO antibody had no evidence of systemic involvement [7]. In a retrospective study from five centers in the Australian Scleroderma Cohort Study (ASCS), 8.9% of 1303 SSc patients were ANCA positive. Autoantibodies (anti-SCL-70 and anti-PR3), interstitial lung disease (ILD), pulmonary embolism, and death events were more common in the ANCA-positive patients. However, only three ANCA-positive patients had confirmed AAV [12]. In addition, anti-MPO-positive SSc patients may have acute kidney damage, which needs to be differentiated from SRC. It remains a great challenge to make a timely and accurate diagnosis; therefore, we need to further clarify the incidence and related significance of AAV in SSc patients.

Only a few studies have reported the clinical manifestations of AAV in SSc cases. Arad et al. described three cases of SSc associated with AAV and reviewed 37 such cases reported in the English literature [9]. It was found that d-penicillamine treatment might be associated with the development of AAV in SSc. Most of the patients were treated with high-dose corticosteroids and cyclophosphamide but had a poor prognosis. Approximately half of the patients improved, 14% progressed to end-stage renal disease requiring renal replacement therapy, and 34% died. AGN usually occurs at least several years (median 7 years) after the initial development of SSc, whereas SRC typically presents within 1–4 years (median: ~ 7–8 months) of SSc diagnosis. AGN differs from SRC in terms of pathological type, such as anti-SCL-70 antibody, blood pressure, incidence of ILD, thrombotic events, kidney replacement therapy, and death rate [13]. It was suggested by another study that SRC generally occurs within 5 years of dcSSc, whereas AAV is usually diagnosed 5 or more years after lcSSc; malignant hypertension was more common in SRC; urine protein was less than 1 g in SRC, whereas AAV caused red blood cell (RBC) cast and proteinuria; anti-RNA polymerase III antibody was frequently detected in SRC while anti-MPO antibody was frequently detected in AAV [8]. The prognosis and outcome of AAV and SRC are significantly different; a renal biopsy is critical for making a differential diagnosis, the results of which help clinicians to take reasonable treatment measures for improving patient prognosis. These differences in clinical manifestation, laboratory tests, and autoimmune antibody (summarized in Table 1) are valuable for the differential diagnosis of AAV from SRC in patients with SSc.

**Table 1:** Differences in clinical manifestation, laboratory test, and autoimmune antibody between patients with SSc + AGN and SRC

	SSc + AGN	SRC
Disease duration	Long, > 4 years	Short, 1–4 years
Characteristic autoantibody	Anti-MPO, anti-PR3	Anti-RNA polymerase III
Anti-SCL-70 antibody	Common (58.8–76%)	Uncommon (21%)
Blood pressure	Normal or slightly higher	Malignant hypertension
Urine protein	Excessive urine protein (red blood cell cast)	Urine protein < 1 g/24 h
Renal biopsy	Crescent formation	Arterial intimal thickening, thrombosis, fibrinoid necrosis, glomerulosclerosis
Prognosis	Rapid progress and high mortality	High rate of renal replacement therapy

The presence of acute renal injury and hypertension is usually suggestive of SRC; however, the rare overlap between AAV and SSc is often ignored by rheumatologists. Several features of AAV that differ from SRC may aid in the differential diagnosis of AAV from SRC [14, 15]. The patient described here had AAV-like clinical features, including a 4-year history of dcSSc, proteinuria, anti-SCL-70 antibody positivity, and no signs of ILD or thrombotic microangiopathy. An early kidney biopsy test was necessary to confirm this patient’s disease. Initially, high-dose glucocorticoids were not used in this case owing to a concern of triggering SRC. After the kidney biopsy confirmed the presence of cellular crescents, the patient was treated with high-dose glucocorticoids, cyclophosphamide, and plasma exchange, and he achieved significant symptom improvement. Although hemodialysis needed to be continued, the general condition of the patient was improved, with a decreased level of peak creatinine, normal blood pressure, reduced levels of inflammatory indicators, and reduced anti-MPO antibody titers. Unlike the case described in a previous report by Uri and his colleagues [13], AAV occurred here in a patient who had not taken penicillamine, suggesting that SSc patients who are not receiving penicillamine treatment may be at a higher risk of developing vasculitis. Moreover, our patient was treated with methylprednisolone for a short time, and his creatinine level was decreased in February before he was transferred to our hospital. Nevertheless, the pathological changes remained unknown in the absence of a renal biopsy, and the patient was not given adequate medication. His kidney function was not recovered to normal range, even with high-dose glucocorticoid plus cyclophosphamide therapy. Lastly, the patient had not had a follow-up visit in the previous 4 years after his diagnosis of SSc. Therefore, the examination of changes in routine test results on blood, urine, and liver function is important to improve the prognosis of patients diagnosed with SSc.

In conclusion, although AAV overlapping with SSc is rare, it does occur and is associated with considerable morbidity and mortality. Therefore, recognizing AAV in the setting of worsening renal function in SSs is critical for providing adequate treatment. Several clinical features can suggest a diagnosis of AAV rather than SRC, but a renal biopsy is required for accurate diagnosis.

## Data Availability

The datasets generated and/or analyzed during the current study are available from the corresponding author on reasonable request.
